# The impact of environmental and dietary exposure on gestational diabetes mellitus: a comprehensive review emphasizing the role of oxidative stress

**DOI:** 10.3389/fendo.2025.1393883

**Published:** 2025-04-02

**Authors:** Congcong Sun, Jiaying Shen, Rujing Fang, Huiya Huang, Yanan Lai, Yanjun Hu, Jianqiong Zheng

**Affiliations:** ^1^ Department of Scientific Research Center, The Third Clinical Institute Affiliated of Wenzhou Medical University, The Third Affiliated of Shanghai University, Wenzhou People’s Hospital, Wenzhou Maternal and Child Health Care Hospital, Wenzhou, China; ^2^ Department of Obstetrics and Gynecology, Wenzhou People’s Hospital, Postgraduate Training Base Alliance of Wenzhou Medical University, Wenzhou, China; ^3^ Department of Intensive Care Unit, The First Affiliated Hospital of Wenzhou Medical University, Wenzhou, China; ^4^ Department of Reproduction and Genetics, The Third Clinical Institute Affiliated of Wenzhou Medical Department of Reproduction and Genetics, The Third Clinical Institute Affiliated of Wenzhou Medical University, The Third Affiliated of Shanghai University, Wenzhou People’s Hospital, Wenzhou Maternal and Child Health Care Hospital, Wenzhou, China; ^5^ Department of Obstetrics and Gynecology, The Third Clinical Institute Affiliated of Wenzhou Medical University, The Third Affiliated of Shanghai University, Wenzhou People’s Hospital, Wenzhou Maternal and Child Health Care Hospital, Wenzhou, China

**Keywords:** gestational diabetes mellitus, oxidative stress, reactive oxygen species, treatment, environmental exposure, dietary exposure

## Abstract

Gestational diabetes mellitus (GDM) is a common pregnancy complication closely associated with maternal oxidative and antioxidant imbalance, known as oxidative stress. Environmental and dietary exposure plays an important role in inducing oxidative stress during pregnancy. This review aims to provide an in-depth analysis of the role of oxidative stress induced by environmental and dietary exposure in GDM while incorporating current research frontiers. Environmental pollution, smoking, excessive nutrition, and unhealthy eating habits such as a high-fat diet and vitamin deficiency, may contribute to the generation and accumulation of reactive oxygen species (ROS), leading to oxidative stress. Within the pathway of oxidative stress in GDM, the production and clearance mechanisms of ROS play a pivotal role. Relevant studies have demonstrated that ROS production is closely linked to insulin resistance, adipose tissue accumulation, inflammation, and other pathological processes. Antioxidant substances like vitamins C and E or glutathione can mitigate oxidative stress damage on pregnant women and fetuses by scavenging ROS. Currently, there remain several cutting-edge issues regarding the involvement of the oxidative stress pathway in GDM pathogenesis as well as its relationship with environmental and dietary factors, for instance: how to reduce maternal oxidative stress levels through dietary adjustments or lifestyle modifications; how antioxidant substances can be utilized for intervention treatment; and accurate assessment methods for maternal oxidative stress status along with its association with GDM risk. In conclusion, environmental and dietary factors exert significant influence on GDM pathogenesis while highlighting increasing attention toward understanding the role played by the oxidative stress pathway within this context. In-depth research endeavors within this field are anticipated to offer novel insights into prevention strategies as well as therapeutic approaches for GDM.

## 1 Introduction

Gestational diabetes mellitus (GDM) refers to abnormal blood glucose levels during pregnancy in women without a prior diabetes diagnosis and is a major cause of childbirth complications (1). Typically, GDM develops during the second or third trimester and is becoming increasingly prevalent worldwide, with rates as high as 20% in China (2, 3).

Compared to diabetes, GDM can cause more complications during pregnancy (4, 5). It can lead to short-term adverse effects in the fetus, such as neonatal respiratory distress syndrome, high bilirubin levels, hypoglycemia, low blood calcium, erythrocythemia, and macrosomia, as well as long-term adverse outcomes, including high weight and obesity in children and adolescents, impaired glucose tolerance, diabetes, and abnormal neurobehavioral development (6). Pregnant women with GDM and their offspring also face a higher risk of developing type 2 diabetes mellitus and deformity disease. Research indicates that diagnosing and treating GDM before 34 weeks of gestation can significantly reduce the likelihood of having a large baby (7, 8). Therefore, early diagnosis coupled with timely treatment plans are highly effective in reducing maternal and infant complications and improving pregnancy outcomes.

Current evidence strongly indicates that environmental and dietary exposures can significantly augment oxidative stress, thereby exerting a profound impact on the development of adverse pregnancy outcomes, including but not limited to GDM. To systematically uncover how environmental and dietary factors affect pregnant women with GDM through oxidative stress mechanisms, we conducted a comprehensive search of relevant studies to date. After analyzing these studies in detail, we found that the influence of environmental and dietary factors on oxidative stress cannot be ignored. For example, being in a polluted environment for a long time, or consuming an unhealthy diet high in sugar and fat for a long time, may increase the level of oxidative stress in the body. When this level of stress exceeds the body’s ability to repair itself, it can lead to adverse pregnancy outcomes such as GDM. With early diagnosis and effective treatment, we can intervene in time to reduce the risk of developing GDM. This not only helps to protect the health of pregnant women but also facilitates the normal development of the fetus and improves the quality of the born population. Overall, the effects of environmental and dietary exposures on oxidative stress and the risk of GDM cannot be ignored. Hence, it is imperative to conduct in-depth research and gain a comprehensive understanding of this field to enhance the prevention and treatment of associated diseases, which not only provide us with an in-depth theoretical basis but also help guide early clinical diagnosis and effective treatment, so as to greatly reduce the risk of GDM.

## 2 The pathogenesis of GDM and its relationship with oxidative stress

### 2.1 Pathophysiological process of GDM

The development of GDM primarily stems from insulin resistance (IR) and an imbalance in insulin secretion (9). During pregnancy, there is a decrease in the body’s sensitivity to insulin, resulting in reduced glucose utilization by the mother and increased hepatic glucose output to meet fetal nutrient demands (10). In response to IR, compensatory insulin secretion occurs. Thus, the occurrence of GDM is attributed to inadequate insulin production by pancreatic β cells, leading to the inability to maintain normal blood sugar levels.

### 2.2 Effects of environmental factors on oxidative stress in GDM patients

#### 2.2.1 Chemical exposure

Studies have demonstrated that prolonged exposure to endocrine-disrupting chemicals (EDCs), such as polychlorinated biphenyls (PCBs), polybrominated diphenyl ethers (PBDEs), phthalates (PAEs), and perfluorinated and poly-fluoroalkyl substances (PFAS) (11), is associated with the development of GDM, disturbance in glucose homeostasis, and an increased risk of GDM (12). EDCs can disrupt normal endocrine signaling in the body, thereby affecting blood glucose balance. For instance, Mia Q. Peng et al. reported that each interquartile range increase in log2-transformed mono(2-ethyl-5-oxohexyl) phthalate, one of EDCs, was associated with 2.4 mg/dL elevation in fasting glucose level, 11.8% increase in fasting insulin levels (13). Research has shown that chronic exposure to organic compounds can lead to metabolic disorders and IR through inflammatory responses and disruption of endocrine function, ultimately activating the peroxisome proliferator-activated receptor (PPAR). PPAR is involved in promoting fatty acid beta-oxidation and antioxidant factors while inhibiting activation of the NF-κB (14–16). Additionally, chemical exposure also affects IR by inducing mitochondrial dysfunction, inhibiting phosphorylation, and activating protein kinase B.

#### 2.2.2 Heavy metals

Existing epidemiological evidence indicates that the prevalence of GDM is associated with exposure to heavy metals such as cadmium (Cd), antimony (Sb), and nickel (Ni) (17–21). Heavy metal exposure primarily disrupts redox homeostasis. Numerous studies have demonstrated that Cd can impair the antioxidant oxidase system, resulting in elevated levels of intracellular and mitochondrial reactive oxygen species (ROS). Moreover, oxidative stress and mitochondrial dysfunction can be induced (22). Wenyu Liu et al. reported that, compared with the bottom tertile, the risk ratios (RRs) for GDM were 1.04 for the middle tertile and 1.36 for the top tertile of Cd levels (19). Fitzgerald et al. discovered that Cd selectively accumulates in pancreatic islets, exerting toxic effects on β cells and thereby exacerbating the risk of GDM (23). In addition to Cd, heavy metals Ni and Sb also were reported to increase the risk of GDM by promoting oxidative stress and inducing pancreatic injury. In multiple-metal models, for each unit increase of ln-transformed urinary Ni or Sb, the risk of GDM increased by 18% (18, 24, 25).

#### 2.2.3 Air pollutant

Studies have shown that air pollution is a major environmental problem and one of the main reasons for the increase in global disease rate (26), among which PM2.5 has the most extensive impact. Seung-Ah Choe et al. found that PM2.5 has a strong correlation with the occurrence of GDM in the third trimester (27). Repeated exposure to PM2.5 can activate nitric oxide synthase (NOS) (28), which up-regulates NO in the blood. NO is an important signaling molecule that can lead to oxidative stress. Excessive NO also promotes the release of inflammatory factors and exacerbates the inflammatory response.

### 2.3 Effects of dietary factors on oxidative stress in GDM patients

#### 2.3.1 High-sugar diet

A multitude of studies have demonstrated a significant association between a high-glycemic diet and GDM (29). Consumption of a high glycemic diet can induce the activation of NADPH oxidase in endothelial cells, leading to increased antioxidant enzymes and enhanced expression of oxidized low-density lipoprotein receptor-1 (LOX-1). Prolonged adherence to a high-glycemic diet may result in excessive lipid deposition, oxidative stress, and inflammation, with oxidative stress being closely intertwined with inflammation. Li Wen et al. discovered that the impact of elevated sugar intake on GDM is also influenced by maternal weight. In non-overweight pregnant women, there exists a noteworthy correlation between high sugar consumption and the risk of GDM; however, no such correlation was observed among overweight women (30).

#### 2.3.2 High-fat diet

Maternal obesity or a high-fat diet increases the incidence of GDM and stillbirth, as well as the risk of metabolic syndrome in offspring (31). The consumption of a high-fat diet during pregnancy can alter the composition of the intestinal microbiome (32), which produces numerous metabolic byproducts that can impact host metabolism, exacerbate oxidative stress and inflammation in women with GDM, reduce insulin sensitivity, and influence the initial gut ecosystem of their offspring.

#### 2.3.3 High-protein diet

Western countries, with their developed animal husbandry and high consumption levels, generally use meat as the main source of protein. In contrast, Southeast Asian countries, due to relatively limited economic conditions and differences in agricultural resources, rely more on plant proteins, such as beans, grains, etc., to meet their daily protein needs. According to Zhou et al., 2755 pregnant women from China typically increase their protein intake as a means of supplementing fetal nutrition. However, their research indicates that adopting high protein and low carbohydrate dietary patterns may elevate the risk of GDM (adjusted OR for quartile 4 v. quartile 1.83; 95% Cl 1.21, 2.79; P trend=0.007) (33). Furthermore, pregnant women who consume animal-based protein are at a higher risk of developing GDM compared to those who follow plant-based protein patterns (34–36). This could be attributed to the fact that the animal protein contains a significant concentration of myoglobin, which is involved in lipid peroxidation (37), thereby exacerbating oxidative stress in pregnant women. Moreover, excessive protein consumption can promote IR and enhance gluconeogenesis, both of which have adverse effects on maintaining normal blood sugar levels.

## 3 The current research status on the association between oxidative stress and GDM

Oxidative stress is associated with GDM, as studies have shown. Mogarekar et al. revealed that the total levels of oxidative stress increased while the levels of vitamin C and NO decreased in maternal plasma (38). Lopez-Tinoco et al. analyzed the relationship between oxidative stress markers and pregnancy outcomes, and demonstrated that the levels of oxidative stress determine the outcome of pregnancy outcomes (39). Liang et al. used a high-fat fed-mouse diabetes model to discover that oxidative stress damages the placental vascular endothelium and leads to vascular complications (40). In hyperglycemia, GDM can lead to chronic hypoxic stress and excessive inflammatory response in the intraplacental vascular endothelial cells (41). Coughlan et al. verified the presence of oxidative stress in GDM placenta and lighted that GDM and type 2 diabetes mellitus display similar pathological changes by examining the relative expression levels of oxidative stress markers in placental tissue samples from GDM and healthy pregnancies (42). Additionally, while monitoring superoxide anion (O_2_-) content in blood vessels following NADH oxidase stimulus monitoring with chemiluminescence, Lund observed that the superoxide anion content in the diabetic rabbit carotid artery was much higher than that in healthy rabbits (43). Interestingly, studies have also shown that oxidative stress disrupts signaling pathways related to glucose regulation, as well as the sensitivity of peripheral tissues to insulin, leading to IR, islet β cell dysfunction, and even islet cell damage and hyperglycemia (44). Overall, these studies indicate that oxidative stress plays a role in the occurrence and development of GDM.

### 3.1 The imbalance between anti-oxidation and oxidation induced by GDM

Each cell produces reactive oxygen species, as well as antioxidants such as catalase (CAT), superoxide dismutase (SOD), glutathione peroxidase (GSH-Px), and vitamin E to eliminate ROS and protect tissues from oxidative damage. Malondialdehyde (MDA), which forms due to the peroxidation of lipids, is the main source of free radicals and is another oxidative stress marker. Zhou et al. demonstrated that mice with GDM exhibit placental oxidative stress during late pregnancy characterized by increased MDA levels and decreased levels of antioxidant enzymes, including SOD, CAT, and GSH-Px (45). Additionally, research has shown that the group with GDM did not display any differences in the total antioxidant capacity of their saliva or plasma compared to the healthy group. However, antioxidants, including uric acid and CAT, were decreased, and oxidative stress markers, including MDA and total oxidative stress, were increased (46, 47). Therefore, GDM patients display varying degrees of oxidative stress in their bodies, and their saliva could be a useful and non-invasive method for estimating oxidative stress levels in GDM populations (46, 48).

### 3.2 The effect of ROS on GDM

ROS are considered to be free radical and non-radical derivatives of oxygen that are generated in response to various stimuli, including hyperglycemia and hyperlipemia. These ROS include hydrogen peroxide (H_2_O_2_), hydroxyl radical (·OH), superoxide anion (O_2_-), and nitric oxide (NO) that can interact with cell membranes and DNA to trigger lipid peroxidation and cell damage (49). ROS, a crucial intracellular messenger, can activate many signal transduction pathways to indirectly cause tissue and cell damage. Women with GDM have been reported to produce excess free radicals and have impaired free-radical scavenging mechanisms (50, 51). It has been shown that the level of ROS is increased in the placental tissue of GDM patients and in a culture of JEG3 placental cells (human choriocarcinoma cells) treated with high glucose, indicating that this may be the primary cause of cell damage and apoptosis during the occurrence and development of GDM (52).

Maternal oxidative stress during pregnancy may impair fetal growth and newborn health. However, low levels of ROS play an important role in childbirth, embryo development and implantation, and placental formation and function. As pregnancy progresses, the levels of antioxidants in a pregnant woman’s body increase to balance oxidation and maintain a healthy pregnancy (51). Therefore, alleviating oxidative stress by increasing ROS scavenging is the main strategy for reducing the complications of GDM.

### 3.3 The influence of lipid peroxide in GDM

LPO can be carried into the blood via lipoproteins, eliciting lipid peroxidation and damaging tissues and the vascular endothelium (53). As shown in animal studies, fatty acid oxidation and a peroxide imbalance are observed in the placental tissue of mice with GDM (54). Other studies also show that increased GDM incidence is closely related to lipid metabolism disorders, and a high LPO concentration can damage the placental vascular endothelium (55). LPO can also lead to vascular endothelial lesions that could result in placental hypoperfusion, leading to a decreased supply of oxygen and blood in the placenta and umbilical cord, which is the main cause of fetal distress and even death in pregnancies with GDM. Thus, when blood sugar levels are under control, there is no increase in LPO, there are no vascular complications (56).

### 3.4 The causes of oxidative stress in pregnant women with GDM

Oxidative stress, a key factor in the occurrence and development of diabetes (51), is closely tied to chronic complications of diabetes, such as diabetic angiopathy and diabetic neuropathy. Many scholars have also suggested that the main mechanisms of GDM leading to oxidative stress are as follows:

#### 3.4.1 Attenuation of antioxidant capacity

During hyperglycemia, the levels of vitamin C, vitamin E, and other antioxidants decrease, and the activities of antioxidant enzymes, including CAT and SOD are reduced due to glycosylation. As a result, the metabolites of oxidases and peroxides are significantly increased (57).

#### 3.4.2 Non-enzymatic saccharification of proteins

In hyperglycemia, non-enzymatic saccharification of proteins generates advanced glycation end products (AGEs) which trigger ROS formation by interacting with their specific receptor (RAGE) (58, 59). AGEs are also involved in lipid peroxidation, which is the main mechanism underlying diabetic vascular complications.

#### 3.4.3 NAD(P)H oxidase

A membrane-bound enzyme that mainly produces O_2_^-^, its expression level is increased in endothelial cells (60). Hyperglycemia activates NAD(P)H oxidase, which increases ROS by modulating the stress-sensitive signaling pathway (58).

#### 3.4.4 Mitochondrial respiratory transmission chain

Mitochondria are the main source of ROS and O_2_-, which are the most prevalent free radicals leading to complications in diabetes (61, 62). In the hyperglycemic state, the number of electron donors in the mitochondrial respiratory chain was increased, along with the production of ROS, leading to increased cell damage and cellular dysfunction.

#### 3.4.5 Self-oxidation

The self-oxidation of glucose increases, producing enediol and dihydroxy compounds, which leads to increased ROS production (63).

### 3.5 The relative pathways involved in oxidative stress induced by GDM

Oxidative stress can stimulate transcription factors including nuclear factor erythroid 2-related factor 2 (Nrf2), NF-κB, and activator protein-1 (AP-1). Additionally, oxidative stress is known to trigger and exacerbate inflammatory responses, and persistent oxidative stress can induce chronic inflammation, which can worsen GDM (51, 64).

#### 3.5.1 Keap1/Nrf2/ARE pathway

Oxidative stress can activate various signaling pathways that involve transcription factors. One of these pathways is the Keap1/Nrf2/ARE pathway. Nrf2 is a transcription factor that helps maintain cellular redox balance (65, 66). And K.L. Milan et al. showed that the activity of Nrf2 was inhibited by miR-142-5p downregulation in GDM placenta, leading to impaired angiogenesis (67). Metformin has been demonstrated to enhance endothelial function and mitigate oxidative stress through the regulation of Nrf2 expression. Under normal conditions, the majority of Nrf2 is localized in the cytosol, where it interacts with the protein Keap1. However, when there is oxidative stress, Keap1 dissociates from Nrf2, and this sensitive degradation mechanism is mainly attributed to the N-terminal Neh2 domain of Nrf2 (68). Allowing Nrf2 to enter the nucleus and activate the expression of its target genes, including *HO-1, SOD, GSH*, and *CAT*, which are also regulated by ARE. The Keap1-Nrf2 system regulates the expression of genes involved in lipid metabolism and plays an important role in maintaining glucose metabolism. It also regulates the expression of genes that encode antioxidant enzymes in pancreatic β-cells (69). Tsehay Abebe et al. found that the activation of the Nrf2 significantly improved insulin sensitivity and reduced glucose intolerance in rats on a high-fat diet (70). Also, research has shown that *in vitro* exposure to PM10 decreases cell viability, and reduces levels of the Nrf2 protein and ATP while increasing malondialdehyde (MDA) levels and mitochondrial reactive oxygen species (ROS). Additionally, whole-body exposure to PM10 induces oxidative stress and disrupts the Nrf2 signaling pathway. These findings further highlight that Nrf2 regulated by environmental and dietary factors in regulating play vital role in metabolic balance and responding to oxidative stress.

#### 3.5.2 The TLR4/MyD88/NF-κB signaling pathway

The TLR4/MyD88/NF-κB signaling pathway plays an important role in inflammation and is activated by Toll-like receptors (TLRs), including TLR4. The activation of TLR4 can lead to oxidative stress by increasing ROS and LPS production, exacerbating inflammation and influencing GDM (71, 72). During inflammation in GDM, TLR4 is combined with MyD88 via the adaptor protein MAL, which leads to the stimulation of NF-κB when MyD88 interacts with IRAK and TRAK6. Inflammatory factors are released when NF-κB translocates to the nucleus (73). Previous studies have demonstrated that TLR4 and NF-κB are increased in GDM pregnancies (74, 75). Inhibition of TLR4 signaling has been shown to stimulate insulin secretion, and downregulation of the TLR4/MyD88/NF-κB pathway can alleviate oxidative stress and decrease inflammatory cytokines during GDM (76).

Furthermore, prolonged environmental exposure to certain concentrations of pollutants can activate the NF-κB pathway and induce physiological dysfunction. In mice exposed long-term to PM2.5, DNA damage was markedly increased, along with significant upregulation of IL-1β, IL-6, TNF-α, and NF-κB p65, leading to toxic effects on the bone marrow. Additionally, a high-carbohydrate diet promotes nuclear translocation of hepatic NF-κB p65 and suppresses sorcin transcription, resulting in enhanced *de novo* lipogenesis (DNL) and intrahepatic lipid accumulation both *in vivo* and *in vitro*. Consequently, both high-fat diets and environmental pollutant exposure influence the NF-κB pathway within the body.

**Figure 1:**
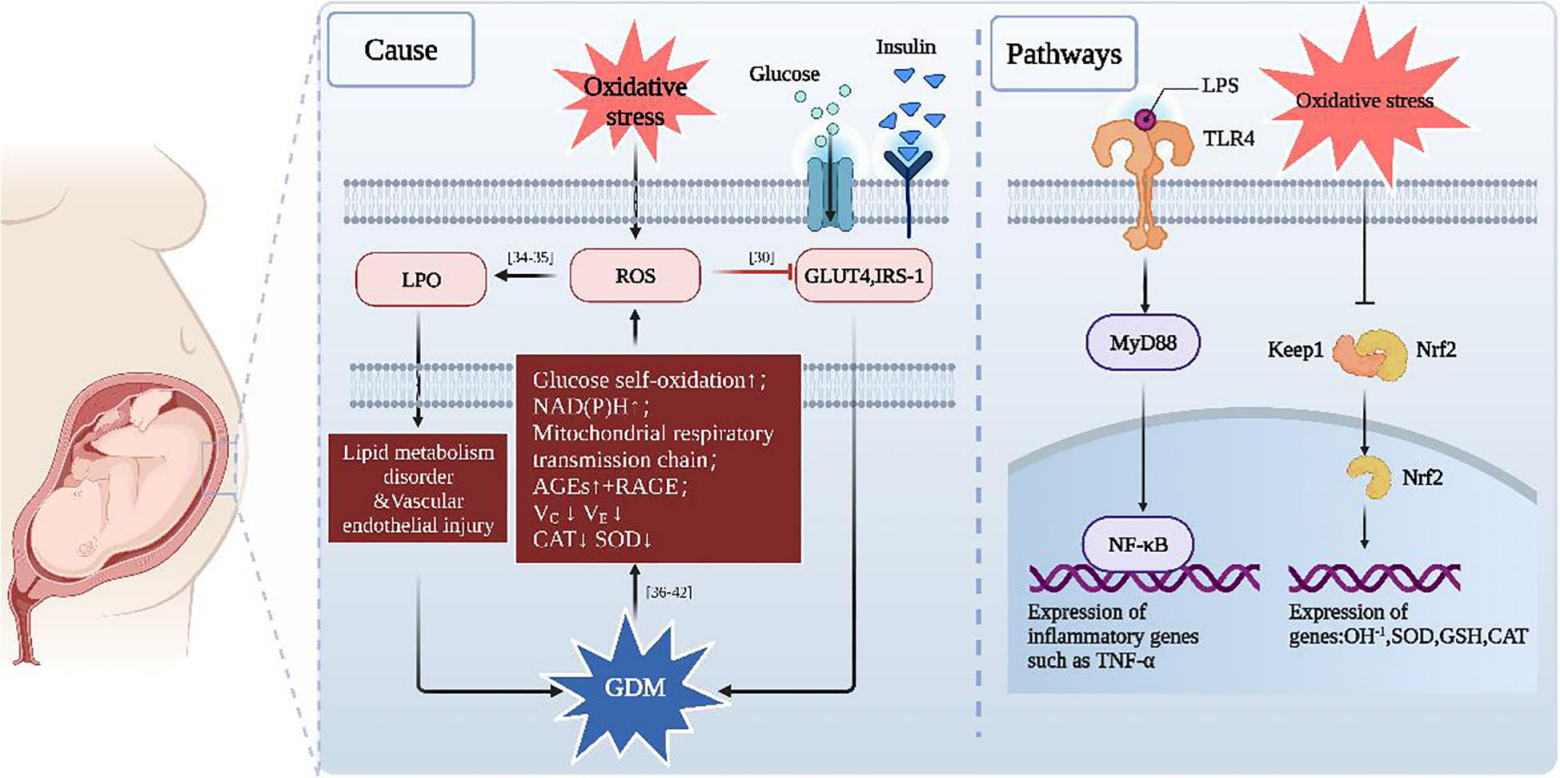
A schematic explaining GDM and oxidative stress. When oxidative stress is increased, ROS and LPO will also increase (55, 77), which will inhibit insulin-stimulated glucose uptake by interfering with both IRS-1 and GLUT4 (50). LPO triggers lipid metabolism disorder and vascular endothelial injury, which can all exacerbate GDM. In addition, GDM leads to excess ROS accumulation through activation of NAD(P) H (78, 79) that increases the self-oxidation of glucose [42], elevates AGEs [38, 39] and mitochondrial respiratory transmission chain (80, 81), while the levels of vitamin C, vitamin E l, CAT and SOD will decrease due to hyperglycemia (82).

As shown in the literature (**Figure 1**), oxidative stress can contribute to many gestational diseases, including GDM. This highlights the importance of maintaining a balance between oxidation and antioxidation as an effective strategy to treat GDM.

### 3.6 The treatment of GDM

#### 3.6.1 The conventional therapies of GDM

**Figure 2:**
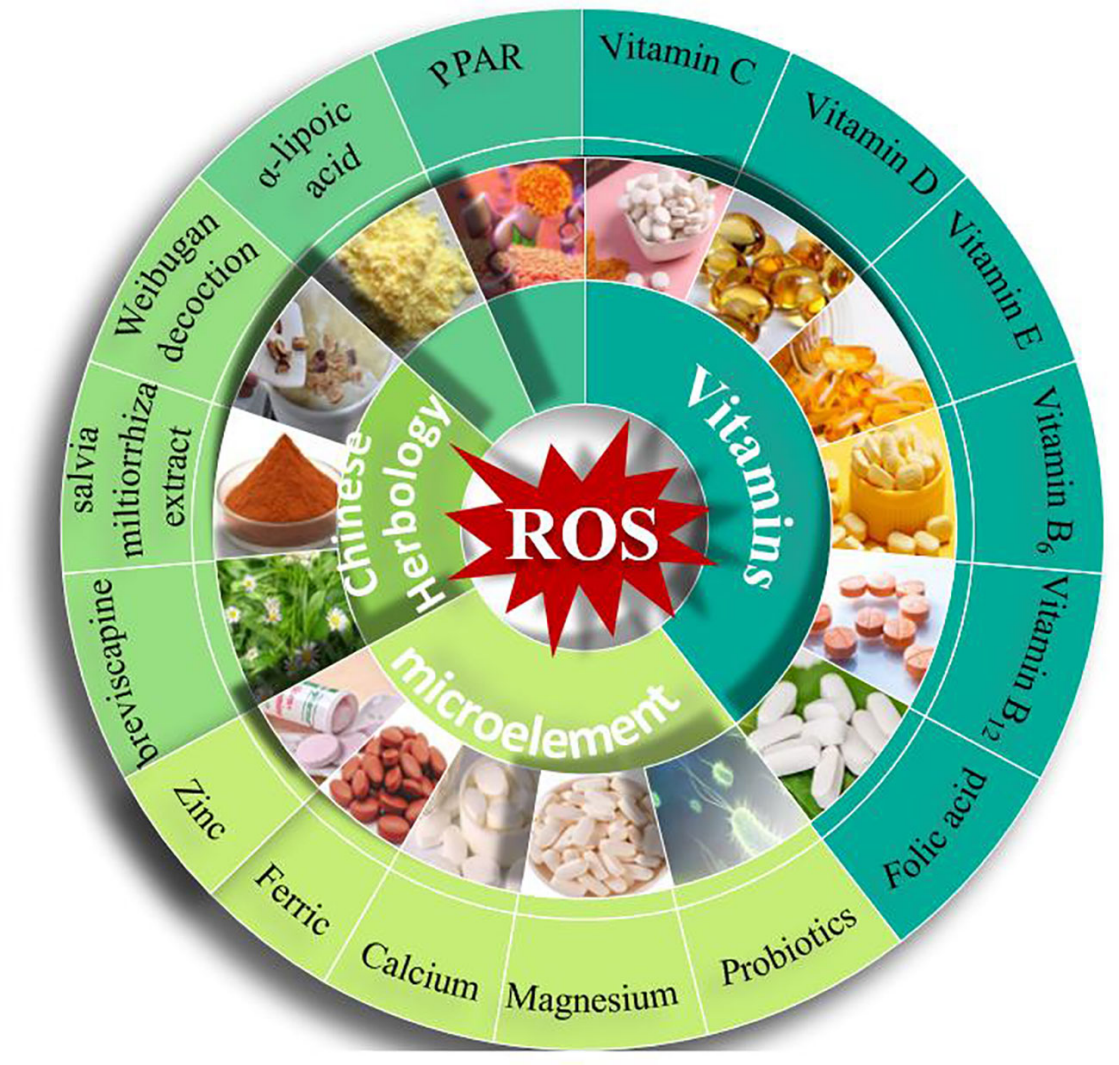
Methods for treating or alleviating GDM.

GDM is a prevalent gestational disease, and as a result, much research has been conducted on its pathogenesis. Currently, the main treatment options for pregnant women with GDM consist of dietary management and lifestyle modifications. In more severe cases, drug therapy may be used in conjunction with these interventions (**Figure 2**).

##### 3.6.1.1 Medical nutritional therapy

GDM regulates their blood sugar levels and prevent complications. These nutritional plans strictly control caloric intake and regulate the consumption of carbohydrates, proteins, lipids, vitamins, minerals, and sugar substitutes. MNT ensures an adequate caloric intake for both the mother and the fetus while preventing excessive weight gain that could lead to related complications (83). Several dietary modifications can effectively lower glucose levels in pregnant women, more so than a standard diet. These modifications include reducing caloric intake for overweight and obese women to approximately 25 kcal per kilogram of body weight. Additionally, limiting carbohydrate content to 35–40% of total calories and focusing on complex carbohydrates instead of simple carbohydrates is recommended. The remaining energy intake should come from proteins (about 20%) and fats (approximately 30-40%), primarily unsaturated. The second modification, in particular, has been shown to improve perinatal outcomes compared to diets that include higher carbohydrate levels (84). It is suggested to have a total of six meals each day, consisting of three main meals and three smaller meals or snacks. Any two consecutive meals should be spaced at least two hours apart and no more than twelve hours apart (85). A diet enriched with extra virgin olive oil (EVOO) has been found to reduce maternal hypertriglyceridemia in GDM pregnancies and to have an anti-inflammatory effect on the placenta (86). Studies show that about 95% of GDM patients can control their blood sugar at ideal levels through simple MNT treatment (87). Combining MNT with physical exercise can also help control pregnancy weight more effectively, as obesity and excessive weight gain are major risk factors for GDM.

##### 3.6.1.2 Pharmacological interventions

Standard drug treatments for GDM include glyburide, metformin, and insulin. Metformin reduces hepatic gluconeogenesis, and intestinal glucose absorption, and increases peripheral glucose uptake and utilization (88). Additionally, metformin alleviates GDM-induced endothelial dysfunction by downregulating p65 and upregulating Nrf2 (74). Glyburide stimulates the release of insulin and lowers blood glucose levels by reducing insulin clearance rate and glucagon secretion in the liver, thereby enhancing the sensitivity of peripheral tissues to insulin (89). Studies show that these treatments, including insulin, are safe and effective for both the mother and the fetus, as they do not induce any differences in childhood growth from 6 months to three years despite growth differences detected at birth (87, 90, 91).

#### 3.6.2 The improvement of GDM caused by oxidative stress

Studies have shown that antioxidant therapy can prevent multiple obstetric complications and improve pregnancy outcomes. The main goals of antioxidant therapy are to reduce oxidative stress (i.e., decrease maternal ROS production), strengthen maternal antioxidant ability, decrease cell apoptosis in amniotic fluid (92–94), and alleviate oxidative damage in the fetus (92).

Antioxidant treatments have been shown to reduce lipid peroxidation and improve the ratio between prostaglandin vasodilators and vasoconstrictors in diabetic placentas, indicating a potential role in adjusting the balance between oxidation and antioxidation in the body. This, in turn, can reduce the generation of LPO in tissues and cells, improve the body’s antioxidant capacity, reverse ischemia and hypoxia, and minimize lesions in the vascular endothelium. Antioxidants have been found to be the most effective treatment against the detrimental effects of GDM on the offspring in animal models of diabetes. However, less research has been conducted on the potential effects of antioxidants on pregnant women with GDM.

##### 3.6.2.1 Vitamins

Studies have suggested that the high incidence of fetal congenital malformation in GDM is mainly due to increased lipid peroxidation and decreased antioxidant capacity. Antioxidants, including vitamins C and E, can reduce lipid peroxidation, enhance the activity of antioxidant enzymes, and alter the activity of fetal SOD and catalase in various tissues to effectively prevent diseases during pregnancy (95). The relationship between vitamin C and GDM is controversial, and different studies have given different insights. A study in Iran uncovered no statistically significant association between serum vitamin C levels and GDM (96). In contrast, a Chinese study demonstrated that the increased risk of GDM from maternal blood exposure to arsenic (As) and mercury (Hg) could be mitigated through vitamin C supplements and high dietary vitamin C intake (97). In addition, more researchers tend to suggest that vitamin C intake can reduce the risk of GDM. The diversity of the results of these studies may be related to the different indicators used by each survey in assessing vitamin C. Moreover, it was verified that vitamins E and C are beneficial for diabetic women with lower concentrations of antioxidants in their plasma; however, the DAPIT (Diabetes and Pre-eclampsia Intervention Trial) studies have revealed that these vitamins do not reduce the occurrence of preeclampsia in pregnant women with type 1 diabetes mellitus without additional treatments (98). The benefits of vitamin supplementation may be limited to women with vitamin deficiency, which needs to be further confirmed by recruiting a large enough population for randomized trials. And in the management of diseases, the administration of vitamins serves primarily to provide an auxiliary anti-oxidative effect and should be used in conjunction with other medications.

Folic acid, vitamin B6, and vitamin B12 are known to effectively lower the levels of total homocysteine in fasting plasma and improve arterial endothelial function. Studies have shown that folic acid reduces the incidence of abnormalities in the embryo yolk sac of animal models with GDM, increases the expression of antioxidant enzymes, and also decreases the expression of genes associated with apoptosis (99). Vitamin D deficiency in pregnant women has been linked to an increased risk of developing GDM, and vitamin D supplementation may be helpful in reversing GDM (100–102). Overall, vitamin supplements could be an effective strategy for treating and preventing GDM, but further research is needed to confirm these findings.

##### 3.6.2.2 α-lipoic acid

α-lipoic acid can directly eliminate ROS and free radicals, chelate metal ions, and regenerate other antioxidants to maintain the balance between oxidation and antioxidation. It is an antioxidant that can protect islet cells from free radical damage and interfere with IR to alleviate oxidative stress. In animal models, it has been shown that lipoic acid minimizes the incidence of fetal neural tube defects and prevents oxidative damage to the placental vasculature, reducing the rate of placental abnormalities (103). Therefore, lipoic acid could play a vital role in preventing the occurrence of GDM and its complications. However, the safety and efficacy of α-lipoic acid need to be further studied (104).

##### 3.6.2.3 Peroxisome proliferator-activated receptors ligand or agonist

PPARs are nuclear transcription factors comprising three subtypes: PPARα, PPARβ, and PPARγ, which play important roles in fat and sugar metabolism, oxidative stress, and inflammation. These receptors exhibit anti-diabetic, anti-inflammatory, and antioxidant properties. Studies have shown that PPAR agonists improve insulin sensitivity and glucose tolerance in diabetic animal models and in women with GDM. They also reduce the lipid peroxidation reaction in the placenta of diabetic mice. A diet rich in PPAR ligands has the potential to prevent NO production induced by hyperglycemia in embryos and reduce the incidence of congenital malformations (105). These findings suggest that PPAR ligands may prevent the overexpression of free radicals, as well as the development of GDM and its complications.

##### 3.6.2.4 Chinese herbology

Antioxidants commonly used in clinical settings cannot completely eliminate oxidative stress. Therefore, there has been increased interest in using traditional Chinese medicine to treat oxidative stress in GDM. Traditional Chinese medicine emphasizes syndrome differentiation and treatment, that is, individualized treatment according to the patient’s physique, illness, symptoms and so on. Breviscapine, a traditional Chinese medicine, has been shown to reduce the amount of LPOs and enhance the effects of antioxidant enzymes. Weibugan decoction is another effective prescription for treating diabetic vascular diseases. Salvia miltiorrhiza extract can also reduce MDA and ROS levels and increase total antioxidant status (TAS) and SOD activity in serum (106). The Lingguizhugan decoction enhances the antioxidant capacity of GDM patients by increasing SOD levels and decreasing MDA content (107). However, these studies lack pharmacokinetic research, such as optimal dosing and bioavailability, as well as reliable clinical trial data to validate their effectiveness. Additionally, certain herbal ingredients may have adverse effects on the fetus, necessitating further clinical research to determine their suitability for treating GDM.

##### 3.6.2.5 Microelement

Iron, a transitional metal, catalyzes the reaction from O_2_^−^ and H_2_O_2_ to the extremely reactive •OH within the mitochondria (108). Ferritin in the serum has been correlated with oxidative stress and GDM (109), indicating that excessive iron intake can also be harmful and associated with oxidative stress in GDM.

Selenium and zinc are trace elements that are necessary for the activity of certain antioxidant enzymes, which may explain why their deficiency is correlated with the incidence of GDM (2, 110, 111). Jamilian et al. reported that magnesium, zinc, and calcium supplements reduce oxidative stress and improve pregnancy outcomes in patients with GDM (102). Selenium has been shown to be involved in maintaining normal glucose uptake, regulating cellular glucose use, and reducing IR. The association between selenium and hyperglycemia in pregnancy may be due to its antioxidant and insulin-mimetic functions (112). Dietary intake and serum levels of zinc are significantly associated with hyperglycemia in pregnancy (113). Zinc can exert multiple indirect antioxidant functions (114), and its deficiency reduces the response to insulin, while its supplementation appears to be beneficial for glucose homeostasis (115). Maternal diabetes has been found to lead to zinc deficiency in fetuses in diabetic rats, and zinc plays an important role in the action of many enzymes and cellular processes, so this deficiency may be one of the teratogenic causes of maternal diabetes (116). It is important to note that dietary intake of zinc and selenium is not the only determinant of their serum levels, as other factors such as age, oxidative stress, chronic disease, and inflammation may also affect their levels. Therefore, the intervention method of trace elements can only alleviate metabolic abnormalities in pregnancy, fetal morbidity, and related adverse consequences, and cannot be used as the main treatment method. Nonetheless, the daily intake of these trace elements should be within recommended limits, and integrating supplements with medications can optimize their efficacy.

##### 3.6.2.6 Lifestyle modifications

In addition to diet management, traditional Chinese and Western medicine, and tonic therapy, maintaining appropriate exercise, as a form of lifestyle modification, is beneficial for pregnant women with GDM in controlling blood glucose levels and alleviating symptoms (117, 118). Exercise, which follows the FITT principle encompassing frequency, intensity, type, and time, refers to regular physical activity tailored to individual needs. The American College of Obstetricians and Gynecologists (ACOG) recommends that pregnant women, barring any medical or obstetric contraindications, should engage in approximately 30 minutes of moderate-intensity aerobic exercise, including activities such as walking, swimming, adapted yoga, Pilates, and stationary cycling, on most days of the week. During physical activity, our muscles utilize glucose from the bloodstream as an energy source independently of insulin. Research has demonstrated that women with GDM exhibit decreased GLUT4 expression in muscle tissue. Nevertheless, engaging in exercise can augment GLUT4 expression and facilitate its translocation from the intracellular compartment to the cell membrane. This process enhances muscle insulin sensitivity and promotes glucose uptake by the muscles independently of insulin’s action. The benefits of exercise persist even after physical activity has ceased, aiding in the maintenance of normal blood glucose levels for extended periods. Furthermore, exercise boosts energy expenditure and improves both insulin sensitivity and glucose tolerance, thereby contributing to effective weight management. Moreover, low-intensity exercise offers advantages for GDM patients. According to Yu et al., exercise can significantly reduce the incidence of GDM (119), thus lowering the risk of adverse pregnancy outcomes, such as macrosomia, premature delivery, and cesarean section (120). Beyond maternal benefits, exercise positively influences fetal development and offspring health throughout their lifespan. Moreover, these healthy habits can also mitigate the risk of cardiovascular disease and type 2 diabetes mellitus in pregnant women with diabetes, thereby enhancing overall maternal health.

##### 3.6.2.7 Probiotic bacteria

In comparison to healthy pregnant women, GDM individuals have a greater abundance of bacteria belonging to the genera Ruminococcus, Eubacterium, and Prevotella and a lower number of bacteria belonging to the genera Bacteroides, Parabacteroides, Roseburia, Dialister, and Akkermansia (121). Alterations in these flora may influence insulin sensitivity and metabolism (122). Therefore, changing the intestinal flora may be able to treat GDM. Probiotic bacteria can maintain the balance of intestinal flora, enhance intestinal barrier function, and regulate immune function by growing and reproducing in the intestine (123). Emerging evidence indicates that probiotics exert a beneficial influence on blood glucose regulation, suggesting their potential as an effective tool for mitigating the incidence of GDM (121). Recent research also highlights that adopting healthy dietary practices, including the consumption of probiotics during pregnancy, can significantly lower the risk of GDM (124).

## 4 Conclusion

Oxidation is a natural part of human metabolism, but an imbalance in antioxidant pathways can lead to excessive free radicals such as ROS and RNS, causing oxidative stress. Several factors can contribute to oxidative stress, including hyperglycemia. Hyperglycemia can trigger the accumulation of ROS by activating NAD(P)H, increasing glucose autoxidation, and altering mitochondrial respiratory transport chains. Furthermore, oxidative stress disrupts signal transduction involved in glucose regulation, exacerbating GDM.

Though the relationship between oxidative stress and GDM is complex, evidence supports the crucial role of oxidative stress in GDM pathogenesis. By focusing on prevention and treatment strategies for oxidative stress, we can reduce the incidence and severity of GDM, and improve outcomes for mothers and infants. Ongoing research in this field is essential for understanding GDM pathogenesis and developing more effective prevention and treatment measures. At the same time, the study revealed that the appropriate exercise plan formulated in accordance with the FITT principle can not only effectively control blood sugar and optimize pregnancy outcomes for pregnant women with GDM, but also comprehensively improve their health level. Exercise plays a multi-dimensional role by enhancing insulin sensitivity, promoting glucose uptake and weight control, and has a positive impact on fetal and offspring health. Therefore, under the guidance of medical professionals, encouraging pregnant women with GDM to implement regular exercise is considered as a safe and effective management means. Biomarkers for early detection of GDM and oxidative stress, may aid in diagnosis and treatment, improving outcomes for mothers and infants. And detection kits for early detection of GDM are expected to be developed, which would allow for predicting the development of GDM before poor glucose tolerance is identified. Developing new drugs for GDM treatment is also important, with a focus on safety, stability, effectiveness, and side effects.

However, the results from these studies may not represent the global population, as most were conducted in China. Additionally, a significant limitation of these studies is the relatively small size of the participant groups. Therefore, future research on GDM women from various geographic locations and with larger sample sizes is necessary to validate these findings. Additionally, Nutritional supplements and probiotics have been shown to decrease biomarkers of inflammation and oxidative stress in laboratory settings and in women with GDM. However, the clinical significance of this reduction, as well as any potential adverse effects on the mother and fetus, remain unclear. As a result, further research is needed to better understand the impact of such supplementation on maternal and fetal health. Meanwhile, the differences in diagnostic criteria among countries significantly affect the diagnosis rate and the heterogeneity of the study population, thus posing challenges to data synthesis. In order to reduce this impact, it is necessary to establish an international consensus to unify diagnostic criteria and standardize research data processing, while strengthening research quality control and using advanced statistical methods to improve the accuracy and reliability of data synthesis. Finally, strengthening international cooperation and exchanges, sharing research data and results, and jointly carrying out research projects are also important ways to promote the in-depth development of gestational diabetes research.
